# Risk of Bronchial Complications After Lung Transplantation With Respiratory *Corynebacteria*. Results From a Monocenter Retrospective Cohort Study

**DOI:** 10.3389/ti.2023.10942

**Published:** 2023-03-01

**Authors:** Adèle Sandot, Nathalie Grall, Thomas Rodier, Vincent Bunel, Cendrine Godet, Gaëlle Weisenburger, Alexy Tran-Dinh, Philippe Montravers, Pierre Mordant, Yves Castier, Philippine Eloy, Laurence Armand-Lefevre, Hervé Mal, Jonathan Messika

**Affiliations:** ^1^ APHP Nord-Université Paris Cité, Hôpital Bichat, Service de Pneumologie B et Transplantation Pulmonaire, Paris, France; ^2^ Université Paris Cité, PHERE UMRS 1152, LVTS UMRS 1148, IAME UMRS 1137, Paris, France; ^3^ AP-HP, Hôpital Bichat, Laboratoire de Bactériologie, Paris, France; ^4^ INSERM, CIC-EC 1425, Hôpital Bichat, Paris, France; ^5^ AP-HP, Hôpital Bichat, DEBRC, Paris, France; ^6^ APHP, Hôpital Bichat, Département d’Anesthésie et Réanimation, Paris, France; ^7^ APHP, Hôpital Bichat, Chirurgie Vasculaire, Thoracique et Transplantation, Paris, France; ^8^ Paris Transplant Group, Paris, France

**Keywords:** lung transplant, infection, chronic lung allograft dysfunction (CLAD), bronchial complications, corynebacteria

## Abstract

*Corynebacterium* spp. are associated with respiratory infections in immunocompromised hosts. A link with bronchial complications after lung transplantation (LTx) has been suggested. We aimed to assess the link between respiratory sampling of *Corynebacterium* spp. and significant bronchial complication (SBC) after LTx. We performed a single center retrospective study. Inclusion of LTx recipients with at least one respiratory *Corynebacterium* spp. sample (July 2014 to December 2018). Subjects were matched to unexposed LTx recipients*.* Primary outcome was SBC occurrence after *Corynebacterium* spp. isolation. Secondary outcomes were *Corynebacterium* spp. persistent sampling, chronic lung allograft dysfunction (CLAD) onset and all-cause mortality. Fifty-nine patients with *Corynebacterium* spp. sampling with 59 without isolation were included. *Corynebacterium* spp. identification was not associated with SBC occurrence (32.4% vs. 21.6%, *p* = 0.342). Previous SBC was associated with further isolation of *Corynebacterium* spp. (OR 3.94, 95% CI [1.72–9.05]). Previous SBC and corticosteroids pulses in the last 3 months were the only factors associated with increased risk of *Corynebacterium* spp. isolation in multivariate analysis. *Corynebacterium* spp. sampling was significantly associated with CLAD onset (27.1% vs. 6.9%, *p* = 0.021). *Corynebacterium* spp. isolation was not associated with SBC but with higher risk of CLAD. Whether CLAD evolution is affected by *Corynebacterium* spp. eradication remains to be investigated.

## Graphical Abstract

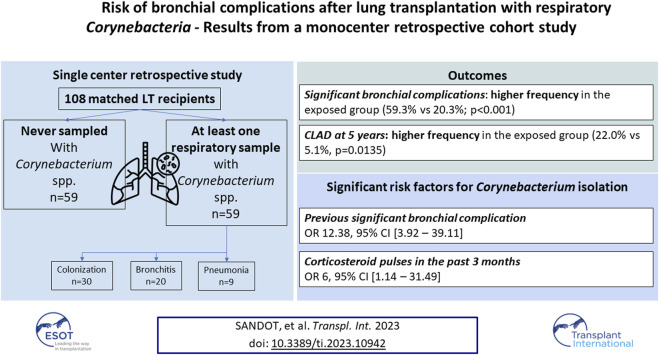

## Introduction

Bronchial complications after lung transplantation (LTx) are a major burden, leading to severe morbidity and mortality (1), occurring in nearly 10% of LTx recipients (LTRs) (2,3). The multiple risk factors might include characteristics of the harvested organ (duration of mechanical ventilation, previous bronchial colonization, duration of cold ischemia) or surgical issues (duration of surgery, anastomosis techniques) (2). Early post-operative complications have also been reported as risk factors for bronchial issues (2). Bronchial complications usually require close bronchoscopic assessment, and in up to 25% of cases (3), interventional bronchoscopy for bronchial stent placement, or balloon dilatation. In a longer perspective, these bronchial complications can lead to functional loss (1). Moreover, repeated respiratory infections and bronchial complications may be linked, as a cause or a consequence (2). For instance, isolation of *Pseudomonas aeruginosa* or *Staphylococcus aureus* has been found associated with bronchial stenosis (4,5). *Corynebacterium* spp. can induce various clinically significant respiratory infections, notably in immunocompromised patients or patients with severe respiratory diseases (6-8). In LTRs, being both immunocompromised and with structural bronchial abnormalities, *Corynebacterium* spp. have been suspected to be associated with bronchial complications (9). In this report, the presence of a bronchial stent was a significant risk factor for persistence of *Corynebacterium* spp. infection.

We aimed to unravel the possible link between respiratory isolation of *Corynebacterium* species and significant bronchial complications (SBCs) in a cohort of LTRs in the Paris-Bichat Lung Transplant Program, France. Our objectives were to investigate the association of *Corynebacterium* spp. isolation and the occurrence of an SBC and to describe the *Corynebacterium* spp. epidemiology, the course of *Corynebacterium* spp. infection and its risk factors and long-term prognosis.

## Patients and Methods

### Patients and Settings

We retrospectively included all adult LTx recipients with at least one lower-respiratory-tract specimen in which a *Corynebacterium* spp. was isolated between July 2014 and December 2018 in the Paris-Bichat Lung Transplant Program. Cases were identified in the local microbiology department database, where all respiratory samples are recorded. Each case was matched to a non-exposed control, selected as the next LTx patient in chronological order. The matching was according to age at LTx ±5 years, mono- or bipulmonary status, underlying respiratory disease (defined in four categories: chronic obstructive pulmonary disease [COPD]/emphysema, interstitial lung disease, bronchial dilatation, miscellaneous). Data were collected anonymously, and the electronic files were used according to French law (*Informatique et Libertés*). The Evaluation Committee for observational research protocols of the French Respiratory Society (SPLF, CEPRO 2020-044) approved the study and waived informed consent.

### Clinical and Microbiological Collected Data

All LTx candidates and recipients have been prospectively included in Paris-Bichat Lung Transplant database since 2006. This database includes demographical and anamnestic data, details on LTx surgery and post-operative course, bronchoscopic findings, and respiratory function.

All lower-respiratory-tract samples (sputum, tracheal aspirate, bronchoalveolar lavage [BAL], or protected distal aspiration) taken during usual care were immediately sent to the bacteriology laboratory. They were inoculated onto routine agar plates incubated for 48 h at 35°C under aerobic and anaerobic conditions. Bacteria were identified at the species level by matrix-assisted laser desorption ionization-time of flight mass spectrometry (MALDI-TOF MS) Microflex LT Biotyper (Bruker Daltonics, Bremen, Germany). Bacterial susceptibility to antibiotics was determined with the disk-diffusion method according to EUCAST guidelines (www.eucast.org).

From the bacteriology laboratory database, we retrieved all cases of LTRs in whom *Corynebacterium* spp. had been isolated in at least one lower-respiratory-tract specimen. In patients with sputum and tracheal aspirates, we included only specimens showing ≥25 leukocytes/field and ≤10 upper respiratory epithelial cells/field, as assessed by the scoring system of Murray and Washington (10). The usual thresholds were applied for interpreting quantitative cultures (i.e., ≥10^4^, ≥10^5^ and ≥10^7^ colony formation units/mL for BAL specimens, tracheal aspiration and sputum culture, respectively). Microbiological data are described in terms of the first *Corynebacterium spp*. isolation.

### LTx Management

Usual management of LTx in our center is highly protocolized and reported elsewhere (11). In brief, intraoperative veno-arterial extra-corporeal membrane oxygenation (ECMO) was initiated according to hemodynamics and respiratory findings during surgery (12), with peripheral cannulation. All patients receive the same initial immunosuppressive regimen (mycophenolate mofetil, corticosteroids and tacrolimus). All patients receive life-long proton pump inhibitors. Antibiotic prophylaxis with cefazoline is administered for 48 h, then adapted to postoperative microbiological analysis. A first bronchoscopy is systematically performed within the first hours after LTx. During the post-operative course, surveillance bronchoscopy and BAL are performed in case of clinically suspected respiratory infection. In case of abnormalities in bronchial healing, these bronchoscopies are repeated, and microbiological samples are taken if an infection is suspected, thus allowing for longitudinal study of colonization.

Transbronchial biopsies are performed in case of clinically suspected acute cellular or antibody mediated rejection (AMR). Acute cellular rejection (ACR) was defined according to established criteria (13), as was AMR (14).

### Study Definitions

We defined an SBC as the presence of a bronchial fistula diagnosed by chest CT-scan or bronchoscopy or the need for interventional bronchoscopy for dilatation or bronchial stenting (2). Persistent respiratory colonization was defined by isolation on a respiratory sample on at least three occasions at least 1 month apart in less than 1 year (15). Chronic lung allograft dysfunction (CLAD) was defined according to ISHLT recommendations (16) as a decline in forced expiratory volume per second (FEV1) ≥ 20% from baseline, persisting at 3-month intervals, excluding other causes. Baseline FEV1 was the mean of the 2 best post-transplant FEV1 measurements. The diagnosis of infection (pneumonia or bronchitis or colonization) was retrospectively defined according to Centers for Disease Control and Prevention definitions (17) by the review of all the medical files by a blinded adjudication committee.

### Study Outcomes

The primary outcome was the occurrence of an SBC. The date of the event was the date of the first SBC. Secondary outcomes were 1) persistent respiratory colonization with *Corynebacterium* spp., 2) CLAD occurrence and its delay after transplantation, 3) all-cause mortality during follow-up, and 4) *Corynebacterium* species and their distribution.

### Statistical Analysis

First, a descriptive analysis was performed in the entire cohort population and according to exposed or unexposed status. Categorical variables were summarized as counts (percentage) and frequency distributions were compared with the Mac Nemar test. Continuous variables were expressed as median (IQR) and differences were tested with the Wilcoxon test.

Second, we compared occurrence of SBC between exposed and unexposed patients in the subset of patients with respiratory *Corynebacterium* spp. isolation before the occurrence of SBC and their matched controls, using the Mac Nemar test. We also searched for factors associated with SBC among the following variables: *Corynebacterium* spp. isolation, underlying respiratory disease, invasive mechanical ventilation duration, ECMO necessity, by a univariate analysis. Third, factors associated with with *Corynebacterium spp*. isolation were investigated by univariate then multivariate logistic regression in the entire cohort population, using the same approach.

Time between LTx and occurrence of CLAD or death was compared between the exposed and unexposed groups by means of survival curves (Kaplan-Meier method) and tested by means of a log-rank test. If the patient was alive or without chronic rejection at the end of the study, the patient was censored at the study end date (December 31, 2018). Those analyses excluded patients with CLAD before isolation of *Corynbacterium* spp. Statistical tests were 2-sided with a significance level of 0.05. All analyses were performed using R software (version 4.0.3).

## Results

### Characteristics of the Cohort

**FIGURE 1:**
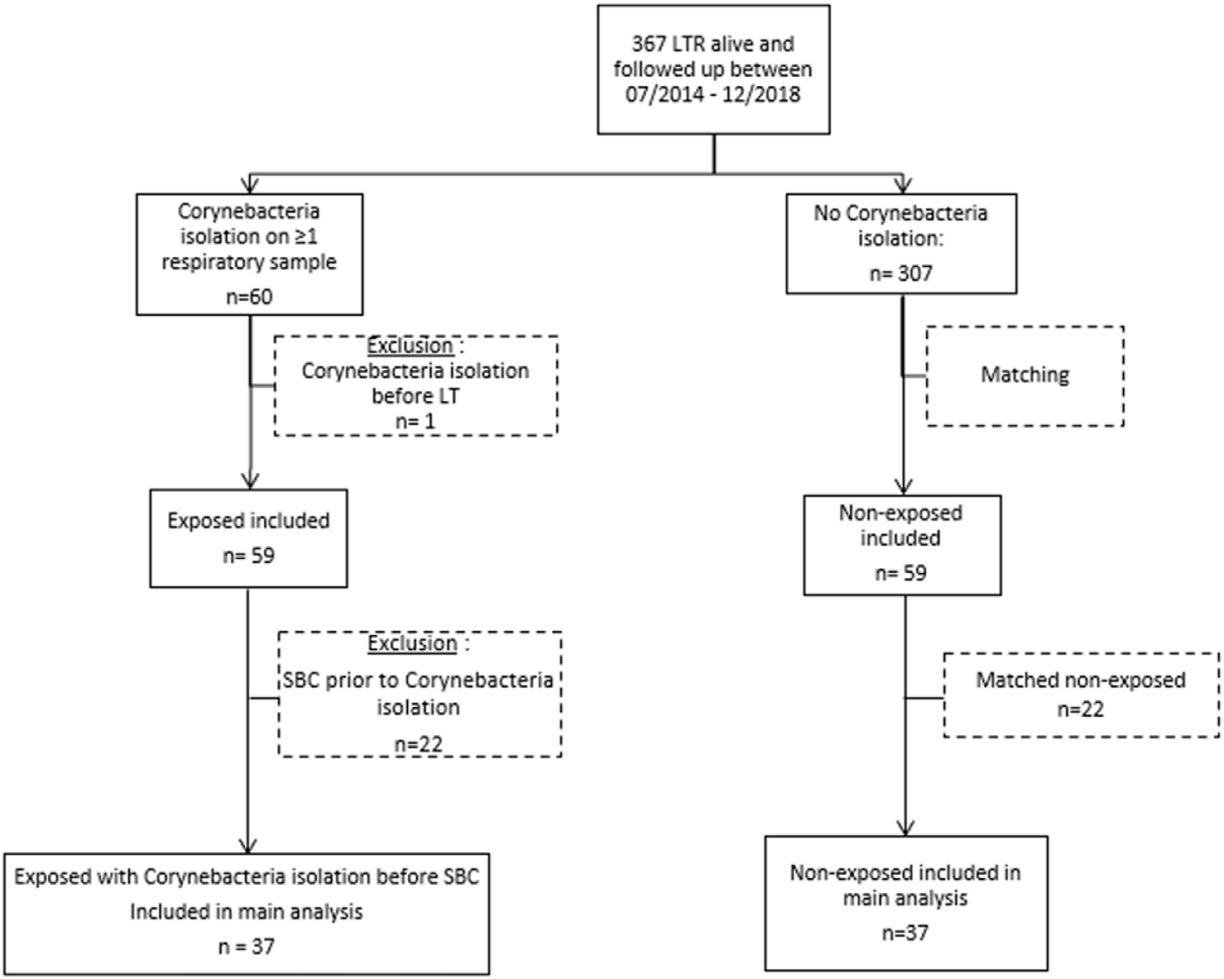
Flowchart of the study. LTR, lung transplantation recipient; SBC, significant bronchial complication.

Over the study period, the cohort of LTx recipient represented 367 patients (Figure 1). *Corynebacterium* spp. were isolated in 60/367 LTRs (16.3% of the cohort) after a median of 128 days [interquartile range 38–503] after LTx. One patient was excluded from the analysis because *Corynebacterium* spp. had also been isolated before LTx on a systematic bronchoscopy, which left 59 LTR as the “exposed” cohort; these were matched to 59 non-exposed LTRs. Patient characteristics are in Table 1.

**TABLE 1:** Baseline characteristics of lung transplantation (LTx) recipients with a lower-respiratory-tract specimen in which a *Corynebacterium* spp. was isolated (exposed) and non-exposed recipients.

	Exposed, *n* = 59	Non-exposed, *n* = 59	*p*-value^a^
Recipient characteristics			
Male sex, n (%)	46 (78.0)	43 (72.9)	0.60
Age (years)	56.1 [50.4–61.5]	56.5 [53.2–59.5]	0.057
Underlying respiratory disease, n (%)			
Emphysema/COPD	25 (42.4)	28 (47.5)	0.25
Interstitial lung disease	28 (47.5)	28 (47.5)	
Bronchiectasis	2 (3.4)	2 (3.4)	
Other	4 (6.9)	1 (1.7)	
Type of lung transplantation n (%)			
Right single-lung	15 (25.4)	17 (28.8)	0.719
Left single-lung	9 (15.3)	8 (13.6)	0.870
Double lung	35 (59.3)	34 (57.6)	1
Highly emergent LTx	7 (11.9)	9 (15.3)	0.75
Intraoperative veno-arterial extracorporeal membrane oxygenation, n (%)	42 (71.2)	31 (52.5)	0.03
Post-operative duration of mechanical ventilation (days)	6.0 [1.0–13.0]	1.0 [1.0–4.0]	0.117
Tracheostomy, n (%)	20 (33.9)	13 (22.0)	0.21
ICU length of stay (days)	16.0 [11.0–34.0]	14.0 [9.5–22.5]	0.521
Primary lung graft dysfunction, n (%)	15 (25.4)	12 (20.7)	0.68
CMV mismatch, n (%)	9 (15.3)	9 (16.1)	1
Immunosuppressive therapy		5	
Corticosteroids	59 (100)	9 (100)	
Corticosteroids dosage^a^ (mg)	20.0 [7.5–30.0]	25.0 [10.0–37.5]	0.18
Mycophenolate mofetil	53 (89.8)	50 (87.7)	1.00
Calcineurins inhibitor	56 (94.9)	56 (98.2)	0.47
Other immunosuppressive therapy in the last 6 months	7 (11.9)	1 (1.8)	0.04
Antimicrobial therapy in the last 3 months	35 (59.3)	31 (52.5)	0.42
Immunological complications			
Acute cellular rejection	13 (22.0)	12 (20.3)	1
Corticosteroids pulses in the last 3 months	24 (40.7)	18 (30.5)	0.26
Allo-immunization or antibody mediated rejection	16 (27.1)	5 (8.5)	0.015

^a^ Prednisone equivalent.

Data are median (interquartile range) unless otherwise indicated.

Baseline data were collected on the date of *Corynebacterium* spp. isolation for exposed patients and on the date with equivalent time to transplantation for non-exposed patients.

Primary lung graft dysfunction was diagnosed according to Snell et al. (43); Acute cellular rejection was diagnosed according to Stewart et al. (13); antibody-mediated rejection was diagnosed according to Levine et al. (18).

Counts presented as n (%); medians presented with interquartile range for non-normally distributed data.

*p*-value for the Wilcoxon or Mc Nemar non-parametric tests as appropriate—logistic regression for categorical variable with more than 2 modalities except for underlying respiratory disease.

COPD, chronic obstructive pulmonary disease; ICU, intensive care unit; CMV, cytomegalovirus.

Strict matching for the underlying respiratory disease leading to transplantation was not possible for 3 cases with rare lung diseases (histiocytosis, lymphangioleiomyomatosis and pulmonary graft-versus-host disease) who were matched with patients transplanted for COPD. Matching on mono- or bipulmonary status was favored whenever possible, to eliminate the risk of confounding colonization or infection of the native lung on isolation of *Corynebacterium* spp.

All patients were receiving systemic corticosteroid therapy, with no difference between exposed and non-exposed patients in median dose of corticosteroids or use of antimetabolites or anticalcineurins. Patients with *Corynebacterium* spp. isolated significantly more frequently received other types of immunosuppressive therapies than non-exposed patients (7/59, 11.9% vs. 1/59, 1.8%, *p* = 0.04; mammalian target of rapamycin inhibitors for 4 patients, belatacept for 2, and rituximab in the previous 6 months for 1 vs. belatacept for 1) (Table 1).

A history of AMR was more common in the exposed than non-exposed group (16/59, 27.1% vs. 5/59, 8.47%, *p* = 0.015) (Table 1). None of the included patients underwent fundoplication surgery.

### Risk Factor for Significant Bronchial Complication

Presence of a SBC was significantly more frequent in the exposed than non-exposed group: 35/59 (59.3%) and 12/59 (20.3%) (*p* < 0.001). Likewise, an interventional bronchoscopy procedure and placement of a bronchial stent were more frequently required in exposed patients (31/59, 52.5% vs. 10/59, 16.9% and 19/59, 32.2% vs. 2/59, 3.4%; both *p* < 0.001). Among the patients with bronchial stents, 12 in the exposed group had a mechanical stent obstruction requiring bronchoscopy and no patient in the non-exposed group.

We analyzed data for 74 patients (Table 2) to evaluate whether *Corynebacterium spp.* was a risk factor for SBC: 21 patients in the exposed group had an SBC before *Corynebacterium spp*. isolation and were excluded from the analysis with their matched control. One non-exposed patient had an SBC before the matched exposed counterpart had *Corynebacterium* spp. isolated. Therefore, he was excluded. The respiratory isolation of a *Corynebacterium* spp. was not associated with increased frequency of further SBC (OR 2.33, IC95 0.60–9.02, *p* = 0.220). The time to onset of SBC did not significantly differ between the two groups. None of each SBC type was significantly more frequent in patients with an *Corynebacterium* spp. isolated.

**TABLE 2:** Factors associated with significant bronchial complication in univariate logistic regression.

	n/N^a^	OR (95% CI)	*p*-value
Previous *Corynebacterium* spp. isolation	37/74	2.33 (0.60–9.02)	0.220
URD - Pulmonary fibrosis	38/74	0.92 (0.17–5.13)	0.928
URD - COPD	34/74	0.88 (0.16–4.99)	0.888
URD - Bronchiectasis	2/74	4.64 (0.04–530.81)	0.526
Intubation length >24 h	63/74	1.01 (0.91–1.12)	0.814
Highly emergent transplantation	11/74	1.85 (0.18–18.91)	0.602
Intra-operative Extra corporeal membrane oxygenation	49/74	1.10 (0.23–5.19)	0.903

^a^ n, frequency; N, number observed; URD: underlying respiratory disease.

### Risk Factors for the Isolation of *Corynebacterium* spp.

We compared data for exposed and unexposed patients by univariate then multivariate logistic regression (Table 3).

**TABLE 3:** Factors associated with *Corynebacterium* spp. sampling in univariate then multivariate analysis.

Covariates	n/N^a^	Univariate analysis			Multivariate analysis		
		Crude OR	CI 95%	*p*-value	Adjusted OR	CI 95%	*p*-value
Previous significant bronchial complication	30/118	10.83	3.47–33.80	<0.001	12.38	3.92–39.11	<0.001
Interstitial lung disease	56/118	1.00	0.49–2.06	1.000			
Emphysema/COPD	53/118	0.81	0.39–1.68	0.579			
Bronchiectasis	4/118	1.00	0.14–7.35	1.00			
CMV mismatch	18/115	0.94	0.34–2.57	0.904			
Tracheotomy	33/118	1.81	0.80–4.11	0.154			
Primitive lung graft dysfunction	27/117	1.31	0.55–3.10	0.544			
Parenteral corticosteroids <3 months	9/118	3.84	0.76–19.30	0.103	6.00	1.14–31.49	0.0034
Antibiotic treatment <3 months	69/118	1.63	0.78–3.42	0.192			
Previous CLAD	6/118	5.37	0.61–47.45	0.131			
Previous AMR	16/118	3.51	1.06–11.62	0.040			
Previous ACR	25/118	1.11	0.46–2.68	0.822			

^a^ n, Frequency; N, number of observation; OR: odds ratio; 95% CI, 95% confidence interval.

COPD, chronic obstructive pulmonary disease; CMV, cytomegalovirus; CLAD, Chronic Lung Allograft Dysfunction (diagnosed accorded to Verleden et al. (16) ACR, acute cellular rejection (diagnosed according to Stewart et al. (13); AMR, antibody mediated rejection (diagnosed according to Levine et al. (18).

Primary lung graft dysfunction was diagnosed according to Christie et al. (19).

The presence of a previous SBC and history of corticosteroids pulses in the last 3 months were the only factors associated with an increased risk of *Corynebacterium* spp. isolation in multivariate analysis (OR 12.38, 95% CI [3.92–39.11]; *p* < 0.001, and 6 [1.14–31.49]; *p* = 0.0034). Prior antibiotic therapy exposure and history of chronic allograft dysfunction were not associated with isolation of *Corynebacterium* spp.

### Clinical Features of *Corynebacterium* spp. Isolation

The presence of *Corynebacterium* spp. in a respiratory sample was associated with a lower respiratory-tract-infection pattern or functional decline in 34/59 (57.6%) LTRs, including 9 (15.3%) with a monomicrobial isolation (Supplementary Table S1). Nine (15.3%) had radiological pneumonia, 2 (3.4%) with monomicrobial isolation. Overall, 20 (33.9%) patients had signs of bronchitis: cough in 14 (23.7%), sputum or bronchoscopic evidence of purulent secretion in 13 (22%) and both in 7 (11.9%). Five (8.5%) had functional decline associated with dyspnea. *Corynebacterium* spp. isolation was associated with functional decline in 14 (24.6%) patients, 5 (8.5%) with monomicrobial isolation. In total, 25 (42.4%) patients had no clinical or biological sign of infection and were therefore considered colonized.

In 42/59 (71%) patients, the first *Corynebacterium* spp. respiratory isolation occurred during the hospital stay, including 14 (24.7%) in the intensive care and 7 during the immediate post-LTx stay. Among the 7 patients admitted to the intensive care unit, 3 (21.4%) had acute respiratory failure and 4 (28.6%) respiratory-related sepsis. Oxygen therapy was needed for 19 (32.2%) patients, invasive mechanical ventilation for 11 (18.6%) patients, and non-invasive ventilation for one patient.

### Description of First *Corynebacterium* spp. Isolation

*C. striatum* was the most frequently retrieved species, accounting for 71.2% of patients (*n* = 42), followed by *C. amycolatum* (14 patients, 23.7%) (Supplementary Table S2); *C. pseudodiphteriticum, C. accolens,* and *C. propinquum* were isolated from one patient each. In 41/59 (69.5%) patients, at least one other bacterium was isolated from the respiratory sample, with 25 (43.1%) above the significance threshold. *P. aeruginosa* was the main bacterium isolated from the plurimicrobial samples, in 18 (30.5%) patients.

### Microbiological Outcomes

In total, 18 (30.5%) patients received effective antimicrobial therapy against *Corynebacterium* spp. infection based on antibiotics susceptibility testing.

Only 31 patients (52.5%) received antibiotic therapy on first isolation; only 18 (58.1%) of these received effective antibiotic therapy for *Corynebacterium* spp. based on antimicrobial susceptibility testing.

Forty-two patients (71.2%) had persistent *Corynebacterium* spp. colonization; among them, 16/18 (88.9%) patients who received an antibiotic course that was deemed effective on *in vitro* data. The antimicrobial therapies are detailed in the Supplementary Table S3. The median duration of *Corynebacterium* spp. carriage was 58 days [interquartile range 7–412]. We found no association between the administration of an effective antibiotic therapy and the presence of persistent colonization.

### Long-Term Outcomes

**FIGURE 2:**
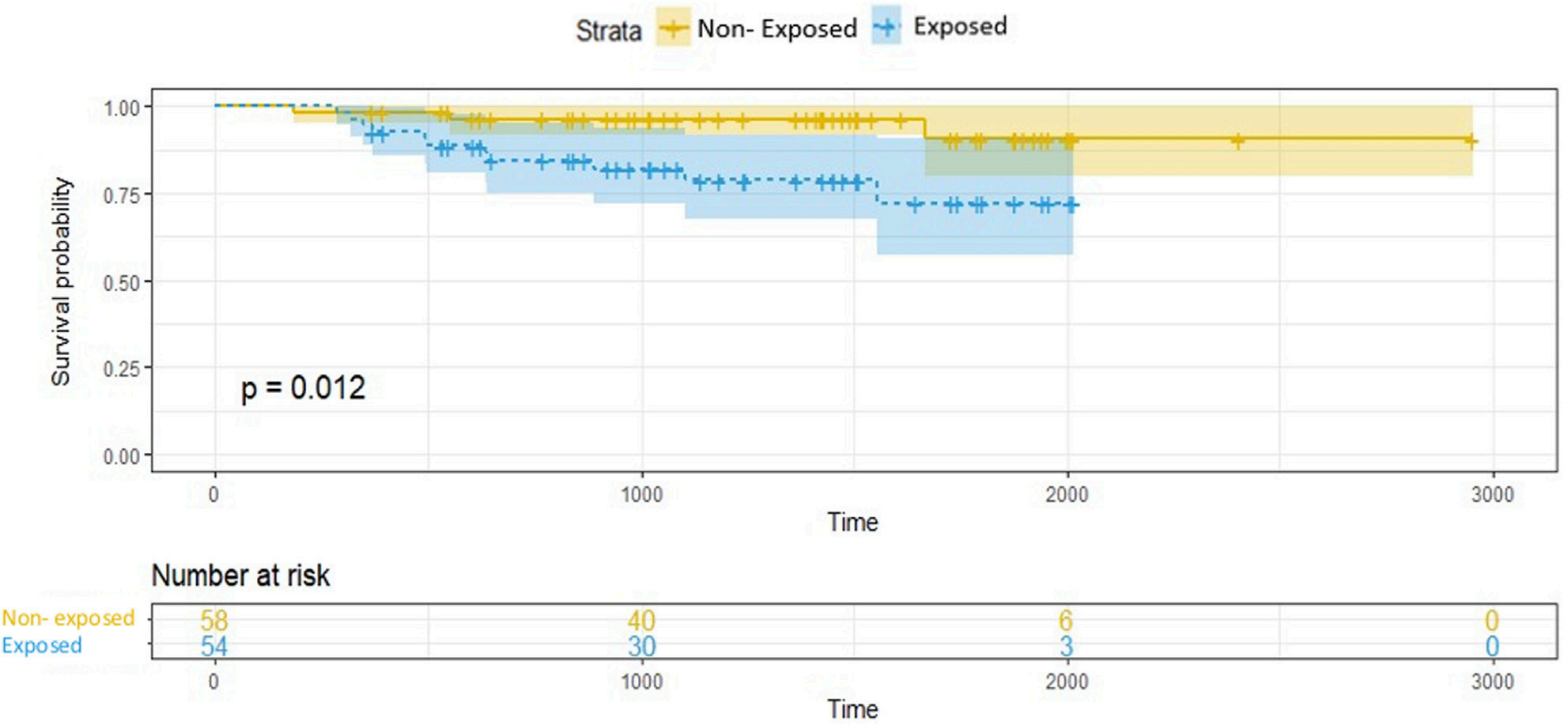
Survival without chronic lung allograft dysfunction. Kaplan-Meier analysis. Survival was measured from the date of transplantation to the end of the collection period. Non-exposed = 0: not exposed to *Corynebacterium* spp.; exposed = 1: exposed to *Corynebacterium* spp.

The frequency of ACR episodes did not significantly differ between the exposed and non-exposed groups. An AMR episode occurred in 16/59 exposed patients, significantly more often than in the non-exposed patients (27.1% vs. 8.5%, *p* = 0.015). The frequency of CMV reactivations did not differ according to *Corynebacterium spp*. exposure (exposed vs. non-exposed 18.6% vs. 8.5%, *p* = 0.178). The length of follow-up did not significantly differ between the groups, but the occurrence of CLAD at 5 years of follow up was significantly higher in exposed than non-exposed patients (27.1% vs. 6.9%; *p* = 0.02122.0% vs. 5.1%, *p* = 0.0135). In the exposed group, CLAD was diagnosed after a median time of 497 days [346–888] of *Corynebacterium* spp*.* first isolation. Conversely, survival without CLAD differed significantly between the two groups (Figure 2; *p* = 0.012), with earlier onset in exposed versus non-exposed patients, but all-cause mortality did not significantly differ.

## Discussion

In this retrospective case–control study, we aimed to investigate the suspected association between *Corynebacterium* spp. isolation in the respiratory tract and the occurrence of an SBC in 118 LTRs. Our findings can be summarized as follows: 1) although a pre-existing SBC was found an independent risk factor for detection of *Corynebacterium* spp., colonization by a *Corynebacterium* spp. was not associated with probability of a subsequent SBC; 2) the presence of a *Corynebacterium* spp. in the lower respiratory tract was associated with clinical manifestations of lower-respiratory-tract infection in 57.6% of cases, 68.6% being associated with another pathogen bacterial species; 3) although 18 (30.5%) patients received an antimicrobial course deemed effective by antimicrobial susceptibility testing, 16 (88.9%) of these had persistent colonization; and 4) survival was higher without CLAD in patients in whom a *Corynebacterium* spp. was never isolated.

The sole series of *Corynebacterium* spp. infection in LTRs reported the course and outcomes of 27 patients with *Corynebacterium* spp. isolation during a 2-year period (9). The low number of patients limits the relevance of the findings. In this series (9), more than half of the patients (53%) had a bronchial complication. The authors defined bronchial complication as the presence of mucosal plaques or purulent secretions at the bronchial suture. Despite guidelines for staging bronchial anomalies (20,21), the description of ischemic bronchitis and its extent is subjective and depends on the evaluator. In our center, fiberoptic bronchoscopies are performed by various physicians (namely LT pulmonologists, general pulmonologists, critical care physicians), not all of them skilled for reporting bronchial complications. A standardized assessment of bronchial complications (20,21) was therefore not available for all the patients. In our work, we deliberately chose an objective, and relevant endpoint, to limit a possible classification bias. Therefore, an SBC was defined as the occurrence of a bronchial fistula or the need for stenting or dilatation. This definition is undisputable as, during the study period, the bronchoscopists with skills on the evaluation of LT bronchial abnormalities were identical, indications for dilatation or bronchial stenting remained unchanged, and all the suspected dehiscence recorded by any physician were confirmed by a single skilled bronchoscopist. We do acknowledge the lack of formal guidelines on the timing or indication of interventional bronchoscopy or the procedure (dilatation or stenting), and the management of these complications may vary between centers. In our center, practices remained unchanged during the study period, with all indications performed by a single expert operator, which highly limits this classification bias. Using this definition, we report that *Corynebacterium* spp. isolation was associated with a pre-existing SBC in 21 (35.6%) patients.

Nevertheless, an interventional bronchoscopy procedure and the placement of a bronchial stent were more frequently needed in patients with *Corynebacterium* spp. isolation. Moreover, the presence of an SBC was an independent risk factor for *Corynebacterium* spp. isolation (OR 12.38 (3.92–39.11, *p* < 0.001).

In the recent study by Los-Arcos et al. (9), the clinical symptoms of lower-respiratory-tract infection were few. Only 12 (50%) patients had signs of respiratory infection (bronchitis, no pneumonia) and 9 when restricted to LTRs with exclusive isolation of *Corynebacterium* spp. and no other pathogen. Data concerning microbiological success or longer-term evolution, especially bronchial evolution, are not reported.

In our series, systemic infection was rare, with only 9 pneumonia (15.3%) cases and 20 (33.9%) with bronchitis.

The isolation of *Corynebacterium* spp. has been reported in immunocompromised hosts [solid-organ transplantation (8), connective tissue diseases under immunosuppressive therapy (22)], and patients with severe underlying respiratory disease (7,23,24). In these settings, infectious episodes related to *Corynebacterium* spp. have quite a silent course, more often appearing as bronchial or tracheobronchial than parenchymal infection (24). In a series of 18 patients with cystic fibrosis (23), 10 (76.9%) had worsening respiratory symptoms and none had pneumonia. In 10 hospitalized patients with COPD (7) 6 had pneumonia and 4 had exacerbations. Of note, in 6 of the 10 samples, the *Corynebacterium* spp. was the sole pathogen isolated and therefore responsible for the clinical symptoms.

In our series, the poor clinical picture may be explained by several combined factors. LTRs receive a high immunosuppressive regimen, thus impairing the T-cell and B-cell response (25-27). Moreover, owing to post-operative anatomical factors, the local host response to infection is decreased (28): modification of bronchial innervation secondary to the surgery decreasing cough reflex (29), impaired lymphatic drainage (30,31) etc.

*Corynebacterium* spp*.* are usually reported as skin and nasal mucosa commensal bacteria. Their isolation in a respiratory specimen is frequently considered a simple colonization, even though several recent studies suggest a varied pathogenicity at different sites (respiratory, endocarditis (32), brain abscess and meningal (33) infections) as well as the possibility of cross transmission of resistant strains between patients (34,35). These factors, associated with a limited number of symptomatic patients, might explain why *Corynebacterium* spp. isolation, even when reaching the microbiological threshold of significance, was not systematically considered to dictate antimicrobial therapy.

The finding of even moderate ischemic bronchitis frequently leads to the prescription of antibiotic therapy, potentially leading to the selection of antibiotic-resistant strains. Among the patients with available antimicrobial susceptibility testing, only 58% had received effective *in vitro* antibiotic therapy (cf. data in supplementary appendix).

In this series, we evidenced an association of *Corynebacterium* spp. isolation following corticosteroids pulses. These results, based on limited effectives (only 9 patients), should be taken carefully. However, these findings are consistent with evidence of increased occurrence of bacterial and fungal infection after immunosuppression intensification (36), and with higher frequency of *Corynebacterium* spp*.* isolation in LTRs receiving other types of immunosuppressive therapies than the conventional immunosuppressive regimen.

We found an association with a previous history of AMR in univariate analysis (OR 3.41 – IC 1.06–11.62), disappearing in multivariate analysis. To our knowledge, the association between respiratory infection or colonization and the occurrence of AMR has not been reported. AMR treatment relies on a heavy immunosuppression regimen (18), which is known to increase the risk of further infection.

Of note, increased risk of ACR has been described after a viral (37, 38) or bacterial (39) infection. In our series, *Corynebac*teriu*m* spp. isolation was not associated with more frequent occurrence of ACR, although ACR is suspected to promote bronchial complications (20).

In our series, the occurrence of CLAD was significantly higher in patients who had at least one positive *Corynebacterium* spp. respiratory sample (27.1% vs. 6.9% in non-exposed patients, *p* = 0.021). Some viral (37) or bacterial (39) lower-respiratory-tract infections or colonization have been reported as risk factors for CLAD (37-39). A single-center retrospective study (40) of 64 patients with post-LTx isolation of *P. aeruginosa* reported a higher frequency of CLAD occurrence within 2 years post-transplantation (23.4% vs. 7.7%, *p* = 0.006) in patients with *P. aeruginosa* colonization. Likewise, another study (41) included 95 LTRs with at least one *P. aeruginosa* isolation. CLAD-free survival was significantly higher in patients with successful eradication than in prolonged colonized patients (*p* = 0.018). These findings support the hypothesis of an inflammatory role of the bacteria, promoting airway damage, and leading to the generation of CLAD (42). Some evidence suggests that a similar mechanism may be involved in *Corynebacterium* spp. infection (42). Obviously, experimental evidence to support these hypotheses are necessary.

Although including a large number of LTRs with a positive *Corynebacterium* spp. lower-respiratory-tract sample, this work has several limitations. This was a single-center study, therefore limiting the significance of its conclusions in other centers. Indeed, in our center, the patients referred for LTx mostly have interstitial lung disease and emphysema. The findings might have been different in a center in which the main underlying respiratory condition would be cystic fibrosis, for example. Nevertheless, the single-center design allows for limiting the confounding factors: the perioperative management and post-LTx follow-up remained identical throughout the study period; the bronchoscopy findings and the indications for endoscopic management of bronchial complications remained unchanged; and the rigorous endoscopic and microbiological follow-up of patients with ischemic bronchitis allowed for reducing the classification bias. All the included LTRs were identified from our center’s microbiology laboratory database. The possibility to have missed the identification of a LTR with a documented *Corynebacterium* spp. in a respiratory sample outside our hospital is unlikely because the management of LTR is highly centralized in our center, for infectious events or for bronchial issues. We decided to match cases and controls according to the underlying respiratory disease, and single or double LTx in order to limit the role of possible pre-existing colonization at LTx. In addition, we referred to published definitions (43) for the various other variables of interest, thus allowing for a homogeneous collection. Because of its retrospective design, neither the susceptibility profile of all Corynebacteria strains nor their phylogenetic relation could be extensively studied.

In conclusion, in this single-center series of 118 LTRs, the isolation of a *Corynebacterium* spp*.* was not associated with a subsequent SBC but occurred more frequently in patients who already had a complication. We found increased frequency and earlier occurrence of CLAD in patients with *Corynebacterium* spp. respiratory isolation. Although we suggest the responsibility of chronic airway inflammation and an association with increased occurrence of AMR, the exact pathophysiology remains to be clarified. The impact of *Corynebacterium* spp. eradication on the occurrence of CLAD should be evaluated in future studies.

## Investigators of the Paris-Bichat Lung Transplant Program

Agnès Abadie, Enora Atchade, Sandrine Boudinet, Pierre Cerceau, Adrian Crutu, Diego Ferreira, Gwenn Frere, Lucie Genet, Tiphaine Goletto, Aurélie Gouel, Sylvain Jean-Baptiste, Gilles Jebrak, Brice Lortat-Jacob, Armelle Marceau, Chahine Medraoui, Lise Morer, Domitille Mouren, Quentin Pellenc, Arnaud Roussel, Mathilde Salpin, Alice Savary, Aurélie Snauwaert, Sebastien Tanaka, Parvine Tashk, Charlotte Thibaut de Menonville, Sandrine Tissot, Sabrina Trigueiros, Nathalie Zappella.

## Data Availability

The raw data supporting the conclusion of this article will be made available by the authors, without undue reservation.
