# Supplementary material for: Risk of Bronchial Complications After Lung Transplantation With Respiratory Corynebacteria. Results From a Monocenter Retrospective Cohort Study
Source: Transpl Int. 2023 Mar 1;36:10942. doi: 10.3389/ti.2023.10942 (PMC10014466; doi:10.3389/ti.2023.10942)
Supplement: Supplementary file 1 [file DataSheet1.docx]

**Risk of bronchial complications after lung transplantation with respiratory *Corynebacteria*. Results from a monocenter case–control series – Electronic supplementary material**

[Author information withheld for double-blind peer review]

**ESM Table 1**. Clinical characteristics at first *Corynebacterium spp.* isolation after lung transplantation (LTx)

|  | **All exposed patients** | **Exposed patients with monomicrobial culture of *Corynebacterium* spp., n=17** |
| --- | --- | --- |
| **Clinical characteristics, n (%)** | | |
| Time to first sampling after LTx (days) (n=59) | 128 [38–503] | 128 [54.50–299] |
| Clinical symptomatology (n=59) | 34 (57.6) | 9 (52.9) |
| Dyspnea (n=59) | 30 (50.8) | 7 (41.2) |
| Clinical infectious symptoms (n=59) | 17 (28.8) | 7 (41.2) |
| Biologic inflammatory syndrome (n=58) | 16 (27.6) | 4 (23.5) |
| Radiographic opacities (n=58) | 9 (15.5) | 2 (11.8) |
| Functional decline (n=57) | 14 (24.6) | 5 (29.4) |

Data are median (interquartile range) or n (%).

Clinical infectious symptoms were defined as fever and/or modification of sputum (increase volume or purulence). Biologic inflammatory syndrome was described as hyperleukocytosis or elevated levels of inflammatory biomarkers C-reactive protein or procalcitonin. Functional decline was described as a decline of more than 10% in forced expiratory volume in 1 sec (FEV_1_) as compared with best personal FEV_1_.

ESM Table 2. Microbiological characteristics of isolated samples

| **Isolated species, n=59** | | |
| --- | --- | --- |
| ***Corynebacterium* spp.** | n (%) | **Bacteria at significance threshold (%)** |
| *C. striatum* | 42 (71.2) | 40 (67.8) |
| *C. amycolatum* | 14 (23.7) | 11 (18.6) |
| *C. accolens* | 1 (1.7) | 0 |
| *C. pseudodiphteriticum* | 1 (1.7) | 0 |
| *C. propinquum* | 1 (1.7) | 0 |
| **Other bacteria in polymicrobial samples** | **N= 41 (69.5)** | **Bacteria at significance threshold (%)** |
| *Pseudomonas aeruginosa* | 18 (30.5) | 10 (17.0) |
| *Staphylococcus aureus* | 5 (8.5) | 5 (8.5) |
| *Escherichia coli* | 3 (5.1) | 3 (5.1) |
| *Stenotrophomonas maltophilia* | 2 (3.4) | 2 (3.4) |
| Other | 13 (22.0) | 10 (16.9) |

ESM Table 3. Antimicrobial therapy at first isolation

|  | **Antimicrobial therapy***  **n=31** | **Adaptation of the antibiotic treatment to the susceptibility testing of the *Corynebacterium* spp. isolate ****  **n=18/31 (58.1%)** |
| --- | --- | --- |
| Beta-lactamin | 24 (77.4) | 12 (38.7) |
| Aminoglycosides | 8 (25.8) | 7 (22.6) |
| Glycopeptide | 2 (6.5) | 2 (6.5) |
| Linezolid | 6 (19.4) | 6 (19.4) |
| Quinolones | 4 (12.9) | 0 |
| Inhaled colimycin | 2 (6.5) | 1 (3.2) |

Data are n (%).

* 15/31 received at least two different antibiotics

** Because of the retrospective design, all susceptibility profiles could not be extensively studied
